# Effects of fat sources on liver characteristics and intestinal morphometry in an early-life animal model

**DOI:** 10.17843/rpmesp.2023.404.12804

**Published:** 2023-12-18

**Authors:** Ivonne M. Gutiérrez Zorrilla, Nataly D. Bernuy-Osorio, Otto Zea Mendoza, Emilio Fredy Yabar Villanueva, Carlos Vílchez-Perales

**Affiliations:** 1 Department of Nutrition, School of Zootechnics, Universidad Nacional Agraria La Molina, Lima, Peru. Universidad Nacional Agraria La Molina Department of Nutrition, School of Zootechnics Universidad Nacional Agraria La Molina Lima Peru; 2 Academic Department of Food Industry Engineering, Faculty of Food Industry Engineering, Universidad Nacional del Centro del Peru, Huancayo, Peru. Universidad Nacional del Centro del Peru Academic Department of Food Industry Engineering Faculty of Food Industry Engineering Universidad Nacional del Centro del Peru Huancayo Peru

**Keywords:** Plant Oils, Fatty Acids, Fatty Acids, Unsaturated, Hepatocytes

## Abstract

We aimed to determine the effect of the consumption of three sources of fatty acids on the relative weight, macroscopic and microscopic characteristics of the liver, and intestinal morphometry in an early-life animal model. Seventy-six randomly distributed chicks received one of the diets (T1: 97.0% basal diet (BD) + 3.0% inert material, T2: 97.0% BD + 3.0% partially hydrogenated vegetable shortening, T3: 97.0% BD + 3.0% quinoa oil, and T4: 97.0% BD + 3.0% fish oil) until the seventh day of life; samples were then extracted in order to be analyzed. We found that the animals that consumed quinoa oil (T3) or fish oil (T4) had favorable results associated to lower liver weight and better absorption of nutrients at intestinal level due to higher values in the hair length and crypt depth ratio, in comparison to partially hydrogenated vegetable shortening (T2). In conclusion, quinoa oil constitutes a healthy option for consumption and an alternative source to fish oil.

## INTRODUCTION

In Peru, malnutrition in children under five years of age persists and coexists with overweight and obesity ^1^. The executive summary on the analysis of the childhood overweight and obesity situation in Peru published in 2023 shows that the prevalence of overweight in children under five years of age is 8.6%, with overweight being the most frequent form of malnutrition; however, the prevalence rate is twice as high in urban areas (10%) when compared to rural areas (4.9%) ^2^. Therefore, overweight and obesity in children under five years of age will influence the development of the infant’s body fat, since it has been shown that obesity is one of the factors that increase the risk of developing non-communicable diseases in adulthood ^3^.

Obese individuals accumulate more body fat than usual, which mainly affects noble organs, such as the liver and small intestine; these organs are susceptible to alterations in their structure and function. These alterations can affect the metabolic functions associated with these organs ^4^^-^^6^. On the other hand, it is important to note that food contains different amounts of lipids, which consist of saturated (SFA) and unsaturated (UFA) fatty acids. In the latter, polyunsaturated fatty acids (PUFA, represented by ω6 and ω3) stand out. Likewise, several studies show that the quality of the fatty acids directly influences adipose tissue, contributing to systemic inflammation, which leads to the search for foods or compounds with high contents of ω3 and ω6; in addition, it has been reported that the consumption of these fatty acids helps in maintaining and/or repairing these organs ^7^.

The consumption of sources of dietary fat may cause different effects on the liver and small intestine depending on their fatty acid content. Therefore, it is interesting to evaluate the impact of the intake of dietary fat sources by Peruvians; these sources include, fish oil (ω3>ω6), which is recommended due to its association with good health but of low accessibility due to its price, quinoa oil (ω6>ω3) as a potential and innovative source, and partially hydrogenated vegetable shortening (AGS>AGI), which is widely used. It should be noted that research this subject has only evaluated the effect of fat sources on the formation of adipose tissue in an animal model ^8^^,^^9^; this is the first study in Peru on the effect of dietary fat sources on the liver and small intestine.

Therefore, this study aimed to evaluate the effect of consumption of three sources of fatty acids (quinoa oil, fish oil and partially hydrogenated vegetable shortening) on the relative weight, macroscopic and microscopic characteristics of the liver, and intestinal morphometry in an animal model at an early age.

**KEY MESSAGES**

Motivation for the study: There is little evidence on whether the consumption of fat sources containing different proportions of fatty acids has an effect on the characteristics of the liver and small intestine at an early age.

Main findings: We found that the intake of fat sources containing unsaturated fatty acids contributes to maintaining the characteristics of both organs; whereas, consumption of sources containing saturated fatty acids favors inflammation in the liver and small intestine.

Implications: The consumption of quinoa oil constitutes an alternative to protect these noble organs in an animal model at an early age.

## THE STUDY

This was a quantitative and experimental research, which lasted seven days.

### Animals

A total of 76 male Cobb 500 chickens, recently hatched, were placed in broiler cages, in an environment at 32 °C and 75% relative humidity. We chose chickens as the experimental animal due to the similarity with humans regarding the deposition of abdominal fat ^10^^-^^12^.

### Sources of fatty acids

Three sources were used: 1) quinoa oil extracted by supercritical fluids (250 bar, 35 °C/60 min, using carbon dioxide as solvent) from ground and sieved Pasankalla quinoa, which was processed at the supercritical fluid extraction pilot plant of the Faculty of Food Industry Engineering, Universidad Nacional del Centro del Perú; 2) fish oil, from salmon (commercial brand); and 3) partially hydrogenated vegetable shortening, commercial brand, purchased from a local market.

Likewise, the medium and long chain fatty acid profile was determined by gas chromatography using a SP2560 100 m × 250 μm × 0.2 μm column with a flow rate of 2.4 mL/min, FID detector at 200 °C and using the standard PUFA 37 mix with different retention times ^13^, from which the PUFA/SFA ratio was obtained from the mentioned sources; their values were 0.22, 3.42 and 2.00 for partially hydrogenated vegetable shortening, quinoa oil, and fish oil, respectively. This ratio suggests that the higher the value obtained, the lower the risk of developing cardiovascular events ^14^.

### Experimental diets

A basal diet was formulated according to the requirements for the first week of life of Cobb 500 chickens (2018), using the Space Animal Nutrition program, more detail can be found in the research by Gutiérrez ^13^. All animals were distributed in four treatment groups with 19 replicates each, they received water and feed *ad libitum* as indicated: i) T1, 97% basal diet (BD) + 3% inert material; ii) T2, 97% BD + 3% partially hydrogenated vegetable shortening; iii) T3, 97% BD + 3% quinoa oil; and iv) T4, 97% BD + 3% fish oil.

The aforementioned diets were prepared daily in order to reduce oxidation and deterioration. In addition, sand was included as an inert material for the control treatment group (T1) so as to not affect the nutrient content regarding the other treatments. At the end of the experiment, each animal was stunned before performing the cervical dislocation for the extraction of liver and jejunum, which were washed with physiological saline solution (at room temperature) until blood remains, intestinal content, etc. were removed; then they were dried with paper towels.

### Relative liver weight

The liver of all animals was weighted on an analytical balance (Model 2204, Digital).

### Macroscopic and microscopic characteristics of the liver

The macroscopic characteristics of the liver were identified by photographs obtained under identical light conditions for each treatment, then four experienced observers used the blind method to evaluate the color, surface roughness and size, which allowed the identification of the degree of the affectation, ranging from grade 0 or normal, I or mild, II or moderate, and III or severe ^15^.

The microscopic structure was analyzed by extracting segments of the greater lobe of the liver for histological preparation according to the standard methodology of embedding in kerosene and staining with hematoxylin-eosin ^16^ with some modifications. Subsequently, the presence/absence of lipid droplets accumulated from each of the histological slides of each treatment was evaluated and analyzed under an optical microscope (Model DM750, Leica) using a 40X objective.

### Intestinal morphometry

Segments of jejunum (approximately 1 cm) were immersed in formalin (10%) for the subsequent preparation of histological slides ^16^. The length, width, villus area and crypt depth of 15 villi for each animal was measured using the Image Analyzer software (Model ICC50W, Leica) of the optical microscope (Model DM750, Leica).

### Statistical analysis

Data were analyzed with the statistical program R Studio version 4.1.1. Normality was evaluated according to the Shapiro Wilk test; liver weight and intestinal morphometry were analyzed by analysis of variance test (ANOVA) and Tukey was used for the comparison of means between treatments.

### Ethical aspects

The research was approved by the Ethics and Research Committee of the Universidad Nacional Agraria La Molina, through report 002-2022-CEI-UNALM; likewise, this study followed the ARRIVE (Animal Research: Reporting of *In Vivo* Experiments) guidelines.

## RESULTS

### Relative liver weight

Significant differences in the average weight of chicken liver were found between treatment groups (Table 1); the animals with a higher average were those that received partially hydrogenated vegetable shortening (T2) compared to the other treatments.

**Table 1:** Relative liver weight, macroscopic characteristics of the liver and intestinal morphometry of chickens fed diets containing different sources of fatty acids.

Variables	T1	T2	T3	T4	p-value
Liver					
Relative weight	5.19^bc^ ± 0.30	5.66^a^ ± 0.53	4.85^c^ ± 0.39	4.93^c^ ± 0.44	< 0.001
Macroscopic characteristics					
Intestinal morphometry					
Length (µm)	399.46^c^ ± 91.8	487.49^b^±105.02	663.71^a^ ±84.38	630.87^a^ ± 71.92	< 0.001
Width (µm)	83.58^ca^ ± 14.01	77.91^cb^ ± 18.01	86.77^a^ ± 16.64	74.21^b^ ± 18.92	< 0.001
Crypt depth (µm)	165.83^a^ ± 177.90	131.45^a^ ±40.56	109.94^b^ ± 28.99	106.59^b^ ± 25.00	< 0.001
Area (mm^2^)	28.227^d^ ± 9.629	33.896^c^ ± 11.707	52.621^a^ ± 14.134	43.046^b^ ± 12.593	0.001
Length/depth of crypt	2.41	3.71	6.04	5.92	-

T1: 97.0% basal diet (BD) + 3.0% inert material, T2: 97.0% BD + 3.0% partially hydrogenated vegetable shortening, T3: 97.0% BD + 3.0% quinoa oil, T4: 97.0% BD + 3.0% fish oil.

a ^-c^: Different letters within the same row indicate significant differences between treatments.

Data were analyzed by analysis of variance (ANOVA) and Tukey for comparison of means between treatments.

### Macroscopic and microscopic characteristics of the liver

**Figure 1:**
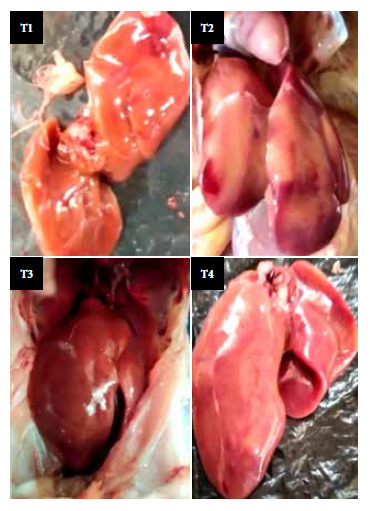
Macroscopic analysis of the liver of Cobb 500 chickens fed with diets containing different sources of fatty acids. T1: 97.0% basal diet (BD) + 3.0% inert material, T2: 97.0% BD + 3.0% partially hydrogenated vegetable shortening, T3: 97.0% BD + 3.0% quinoa oil, T4: 97.0% BD + 3.0% fish oil.

**Figure 2:**
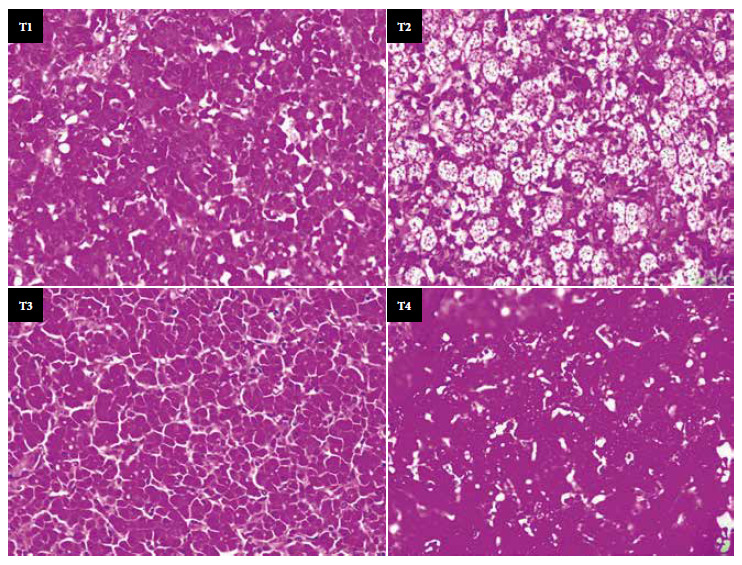
Microscopic analysis of the liver of Cobb 500 chickens fed with diets containing different sources of fatty acids. T1: 97.0% basal diet (BD) + 3.0% inert material, T2: 97.0% BD + 3.0% partially hydrogenated vegetable shortening, T3: 97.0% BD + 3.0% quinoa oil, T4: 97.0% BD + 3.0% fish oil.

The macroscopic characteristics of the liver revealed the degree of affectation. The liver of the animals that received T2 (partially hydrogenated vegetable shortening) showed a pale, dull color in most of the organ. In contrast, the liver of the animals that received quinoa oil or fish oil presented normal macroscopic characteristics (Figure 1), which were wine red color, its characteristic brightness and the absence of spots; similar to the control. The microscopic analysis showed that the animals that received the T2 diet had a high accumulation of fat droplets due to the effect of the consumption of partially hydrogenated vegetable shortening (Figure 2), on the other hand, the group of animals that consumed quinoa oil, fish oil or the control diet, showed less accumulation of fat droplets.

### Intestinal morphometry

The animals who received treatment supplemented with partially hydrogenated vegetable shortening (T2) showed lower length, width and area of the villi with respect to both oils (T3 and T4) and greater crypt depth compared to T3 and T4 (p<0.05) and a value similar to the basal diet. Quinoa oil supplementation (T3) showed higher values for intestinal villi length, width and area, but lower values for crypt depth; followed closely by the treatment that received fish oil (T4; p<0.05), which reported similar values for crypt length and depth but lower values for villi width and area.

The relationship between intestinal villi length and crypt depth in treatments supplemented with quinoa oil (T3: 6.04) or fish oil (T4: 5.92) had higher values, compared to those that consumed partially hydrogenated vegetable shortening (T2: 3.71).

## DISCUSSION

It is interesting to understand the effect that fat sources have on liver characteristics and small intestinal morphometry in a young animal model. The liver is the main place where *de novo* lipogenesis occurs, which is a form of cross-communication between the organ and lipids for maintaining metabolic homeostasis ^17^. Our research showed that the animals that consumed partially hydrogenated vegetable shortening (T2) had a higher relative liver weight and were mildly affected (degree I), suggesting the onset of a possible fatty liver; this is probably due to the increase in lipogenesis ^18^^)^ and the presence of SFA in the diet that are associated with a greater accumulation of intrahepatic triglycerides ^19^. The suggested mechanism is based on the fact that SFA consumption increases inflammation in adipose tissue, and consequently, promotes lipolysis of the tissue, leading to a greater flow of fatty acids (dietary and adipose tissue) to the liver and increasing ceramides, which apparently cause insulin resistance, leading to the accumulation of intrahepatic triglycerides ^19^. Previous studies report that the influence of high SFA consumption on the development of liver inflammation can worsen if consumption of SFA becomes continuous, which would lead to the development of fatty liver ^7^^,^^20^. In contrast, there is evidence that the consumption of IFA (ω3 and ω6) decreases and/or prevents the accumulation of intrahepatic triglycerides compared to SFA ^19^.

The contrast with the microscopic analysis indicates that the animals that consumed quinoa oil (T3), fish oil (T4) or the control diet (T1), accumulated less fat due to the effect of PUFA on β-oxidation; likewise, these also influence the increase of anti-inflammatory adipokines such as Interleukin-10, which is widely related to the metabolic syndrome due to its capacity to inhibit the synthesis of inflammatory cytokines ^21^. It has even been determined that the consumption of palm-based oils at 10% increases the activity in the blood vessels of the liver, promoting inflammation of this organ, but it is possible that in the present study, the intake of the T2 diet, which contained partially hydrogenated vegetable shortening at 3%, affected this organ ^22^.

Regarding intestinal morphometry, the crypt length/depth ratio shows that the consumption of quinoa oil (T3) followed by fish oil (T4), promotes higher values that are directly related to an improvement in digestion and absorption of nutrients due to their PUFA content ^23^^,^^24^, compared to the consumption of partially hydrogenated vegetable shortening (T2) and the basal diet (T1). Previous research suggests that, fatty acid composition modulates first, the rate of intestinal digestion and then serum lipid levels ^25^, and even the consumption of vegetable oils at a level ≥6% affects intestinal absorption and promotes the formation of a thin submucosal layer, which would reduce the expansion of the intestine when a large volume of food is ingested ^26^. On the other hand, it has been evidenced that the consumption of PUFA-rich oils can decrease the transport of endotoxins, in contrast, the intake of SFA increases the transport of endotoxins ^27^.

Among the limitations of this study, we should mention that more specific analyses were not performed to determine whether or not there was development of fatty liver in the group that received partially hydrogenated vegetable shortening. Likewise, although the animal model accumulates fat in a similar way to the human one, it is not possible to infer this response, nonetheless, it is necessary to study it due to the findings and their relationship with health.

In view of the above, red quinoa oil is a beneficial fat source due to its capacity to favor the formation of adipocytes related to healthy adipose tissue in the organism. This research is a basis for future studies regarding the impact on the metabolic function of other organs and health, as well as its potential as an ingredient in the preparation of products for human consumption with the aim of benefiting the health of the population.

In conclusion, the consumption of fat sources such as quinoa oil and fish oil reduced the relative weight and maintained adequate macroscopic-microscopic characteristics of the liver, in addition, it favored a better morphometry of the intestine in a young animal model. On the contrary, partially hydrogenated vegetable shortening caused an increase in the weight of the organ, altered its macroscopic and microscopic structure and lowered the absorption capacity at intestinal level. This suggests that quinoa oil has great potential to be included in the diet for human consumption.
