# Supplementary material for: Effects of fat sources on liver characteristics and intestinal morphometry in an early-life animal model
Source: Rev Peru Med Exp Salud Publica. 2023 Dec 18;40(4):459–65. doi: 10.17843/rpmesp.2023.404.12804 (PMC11139498; doi:10.17843/rpmesp.2023.404.12804)
Supplement: Translation

# Translation: Efectos de fuentes de grasa sobre características del hígado y la morfometría intestinal en un modelo animal a temprana edad

**Authors:** Ivonne M. Gutiérrez Zorrilla, Nataly D. Bernuy-Osorio, Otto Zea Mendoza, Emilio Fredy Yabar Villanueva, Carlos Vílchez-Perales

**Affiliations:** 1 Departamento de Nutrición, Facultad de Zootecnia, Universidad Nacional Agraria La Molina, Lima, Perú.; 2 Departamento Académico de Ingeniería en Industrias Alimentarias, Facultad de Ingeniería en Industrias Alimentarias, Universidad Nacional del Centro del Perú, Huancayo, Perú.

**Keywords:** Aceites de Plantas, Ácidos Grasos Saturados, Ácidos Grasos Insaturados, Hepatocitos

## Abstract

El objetivo fue determinar el efecto del consumo de tres fuentes de ácidos grasos sobre el peso relativo, caracterización macroscópica y microscópica del hígado, y la morfometría intestinal en un modelo animal a temprana edad. Se utilizaron 76 pollitos distribuidos al azar, que recibieron una de las dietas (T1: 97,0% de dieta basal (DB) + 3,0% de material inerte, T2: 97,0% de DB + 3,0% de manteca vegetal parcialmente hidrogenada, T3: 97,0% de DB + 3,0% de aceite de quinua, y T4: 97,0% de DB + 3,0% de aceite de pescado) hasta el séptimo día de vida; luego se extrajeron muestras para los análisis correspondientes. Se encontró que, los animales que consumieron aceite de quinua (T3) o aceite de pescado (T4) tuvieron resultados favorables asociados a un menor peso del hígado y, una mejor absorción de nutrientes a nivel intestinal debido a mayores valores en la relación longitud del vello y profundidad de cripta; en comparación a la manteca vegetal parcialmente hidrogenada (T2). En conclusión, el aceite de quinua constituye una opción saludable de consumo y fuente alternativa al aceite de pescado.

## INTRODUCCIÓN

En Perú, en los niños menores de cinco años, aún persisten los problemas de desnutrición coexistiendo con el exceso de peso, que comprende el sobrepeso y obesidad ^1^. El resumen ejecutivo sobre el análisis del panorama del sobrepeso y obesidad infantil en Perú publicado en el 2023 reporta que la prevalencia de exceso de peso en menores de cinco años es de 8,6%, siendo el sobrepeso, la forma más frecuente de malnutrición; sin embargo, en áreas urbanas (10%), esta prevalencia es el doble respecto a las rurales (4,9%) ^2^. Por lo tanto, la condición de sobrepeso y obesidad en menores de cinco años influirá en el desarrollo de la grasa corporal del infante, ya que se ha evidenciado que la obesidad constituye uno de los factores que incrementan el riesgo de desarrollar enfermedades no transmisibles en la edad adulta ^3^.

En un individuo obeso, se observa una mayor acumulación de grasa corporal, que afecta principalmente a órganos nobles, como el hígado e intestino delgado, los cuales son susceptibles a presentar alteración en su estructura y función. Esta alteración puede afectar las funciones metabólicas asociados a estos órganos ^4^^-^^6^. Por otro lado, es necesario resaltar que los lípidos se encuentran en diferentes proporciones en los alimentos y están formados por ácidos grasos: saturados (AGS) e insaturados (AGI). En este último, destacan los ácidos grasos poliinsaturados (AGPI, representados por los ω6 y ω3). Asimismo, diversos estudios sostienen que la calidad de los ácidos grasos consumidos influye directamente en el tejido adiposo, contribuyendo a la inflamación sistémica, lo que propicia la búsqueda de alimentos o compuestos con elevado contenido de ω3 y ω6, además, se ha evidenciado que el consumo de estos ácidos grasos es capaz de mantener y/o reparar estos órganos ^7^.

El consumo de fuentes de grasa causaría diferentes efectos sobre el hígado e intestino delgado en función a su contenido de ácidos grasos, resultando interesante evaluar el impacto de la ingesta de fuentes ingeridas en la dieta peruana, por ejemplo, el aceite de pescado (ω3>ω6) que, es el más recomendado por su asociación con una buena salud pero de baja accesibilidad debido a su precio; el aceite de quinua (ω6>ω3) como fuente potencial e innovadora; y la manteca vegetal parcialmente hidrogenada (AGS>AGI) que es ampliamente utilizada. Cabe señalar que, investigaciones relacionadas solo han evaluado el consumo de fuentes de grasa sobre la formación de tejido adiposo en este modelo animal ^8^^,^^9^, siendo este el primer estudio realizado en el país sobre su efecto en el hígado e intestino delgado.

Por lo tanto, el objetivo del presente estudio fue evaluar el efecto del consumo de tres fuentes de ácidos grasos (aceite de quinua, aceite de pescado y manteca vegetal parcialmente hidrogenada) sobre el peso relativo, caracterización macroscópica y microscópica del hígado, y la morfometría intestinal en un modelo animal a temprana edad.

**MENSAJE CLAVE**

Motivación para realizar el estudio: Poco se sabe si el consumo de fuentes de grasa que contienen diferentes proporciones de ácidos grasos tiene un efecto sobre las características adecuadas del hígado e intestino delgado, en edad temprana.

Principales hallazgos: La investigación encontró que la ingesta de fuentes de grasa que contienen ácidos grasos insaturados contribuye a mantener las características de ambos órganos; mientras que, el consumo de fuentes con ácidos grasos saturados promueve el desarrollo de inflamación en hígado e intestino delgado.

Implicancias: El consumo de aceite de quinua constituye una alternativa para proteger estos órganos nobles en el modelo animal a temprana edad.

## EL ESTUDIO

La investigación es de tipo cuantitativa con un diseño experimental, que tuvo una duración de siete días.

### Animales

Se empleó un total de 76 pollitos machos Cobb 500, recién eclosionados que fueron colocados en jaulas para pollos, en un ambiente a 32 ºC y 75% de humedad relativa. La elección del animal de experimentación responde a la similitud en la deposición de grasa corporal a nivel abdominal como ocurre en el humano ^10^^-^^12^.

### Fuentes de ácidos grasos

Se utilizaron tres fuentes: 1) aceite de quinua extraído mediante fluidos supercríticos (250 bar, 35 °C/60 min, utilizando dióxido de carbono como disolvente) a partir de quinua *Pasankalla*, molida y tamizada, lo que fue realizado en la planta piloto de extracción de fluidos supercríticos de la Facultad de Ingeniería en Industrias Alimentarias, Universidad Nacional del Centro del Perú; 2) aceite de pescado, marca comercial proveniente del salmón; y 3) manteca vegetal parcialmente hidrogenada marca comercial, adquirida del mercado local.

Asimismo, se determinó el perfil de ácidos grasos de cadena mediana y larga mediante cromatografía de gases mediante una columna SP2560 100 m × 250 μm × 0,2 μm con un flujo de 2,4 mL/min, detector FID a 200 °C y utilizando el estándar PUFA 37 mix con diferentes tiempos de retención ^13^, a partir del cual se obtuvo la relación AGPI/AGS de las fuentes mencionadas, cuyos valores fueron 0,22, 3,42 y 2,00 para la manteca vegetal parcialmente hidrogenada, aceite de quinua, y aceite de pescado; respectivamente. Esta relación sugiere que, a mayor valor obtenido menor será el riesgo de desarrollar eventos cardiovasculares ^14^.

### Dietas experimentales

Se formuló una dieta basal, según la tabla de requerimientos de la primera semana de vida de pollitos Cobb 500 (2018), mediante el programa *Space Animal Nutrition*, mayor detalle se indica en la investigación de Gutiérrez ^13^. Todos los animales fueron distribuidos en cuatro tratamientos con 19 repeticiones cada uno, estos recibieron agua y alimento *ad libitum* según se indica: i) T1, 97% de dieta basal (DB) + 3% de material inerte; ii) T2, 97% de DB + 3% de manteca vegetal parcialmente hidrogenada; iii) T3, 97% de DB + 3% de aceite de quinua; y iv) T4, 97% de DB + 3% de aceite de pescado.

Las dietas mencionadas fueron preparadas diariamente con la finalidad de reducir la oxidación y deterioro de las mismas. Además, para el tratamiento control (T1) se consideró la inclusión de arena como material inerte para no afectar el contenido de nutrientes respecto a los demás tratamientos. Al finalizar el experimento, cada uno de los animales de cada tratamiento fue aturdido antes de realizar la dislocación cervical para la extracción de hígado y yeyuno, que fueron lavados con suero fisiológico (a temperatura ambiente) hasta eliminar restos de sangre, contenido intestinal, etc.; y finalmente fueron secados con papel toalla.

### Peso relativo del hígado

Se registró el peso del órgano en mención en una balanza analítica (Modelo 2204, Digital), de cada uno de los animales que recibieron los diferentes tratamientos.

### Caracterización macroscópica y microscópica del hígado

En la caracterización macroscópica, se realizaron tomas fotográficas del órgano en idénticas condiciones de luz para cada tratamiento, luego mediante el método ciego, cuatro observadores con experiencia en el análisis realizaron una valoración clínica del color, rugosidad de la superficie y tamaño, lo que permite establecer el grado de afectación del órgano que va desde grado 0 o normal, I o leve, II o moderado, y III o grave ^15^.

Para analizar su estructura mediante microscopia, se extrajo segmentos del lóbulo mayor del hígado para la preparación histológica según la metodología estándar de inclusión en parafina y tinción con hematoxilina-eosina ^16^ con algunas modificaciones. Posteriormente, se evaluó presencia/ausencia de gotas de lípidos acumulados de cada una de las láminas histológicas de cada tratamiento, siendo analizados en el microscopio óptico (Modelo DM750, Leica) utilizando el objetivo 40X.

### Morfometría intestinal

Los segmentos del yeyuno (1 cm aproximadamente) fueron sumergidos en formol (10%) para la posterior preparación de láminas histológicas ^16^. A partir de estas láminas, se realizó la medición de longitud, ancho, área de vellosidad y profundidad de cripta; de un total de 15 vellosidades para cada animal de cada tratamiento mediante el software Analizador de Imágenes (Modelo ICC50W, Leica) del microscopio óptico (Modelo DM750, Leica).

### Análisis estadístico

Los datos fueron analizados por el programa estadístico R Studio versión 4.1.1. Se evaluó la normalidad según la prueba de Shapiro Wilk, donde, el peso de hígado y la morfometría intestinal fueron analizados mediante la prueba de análisis de varianza (ANOVA) y Tukey para la comparación de medias entre tratamientos.

### Aspectos éticos

La investigación fue aprobada por el Comité de Ética e Investigación de la Universidad Nacional Agraria La Molina, mediante el informe 002-2022-CEI-UNALM; asimismo, este estudio siguió las directrices de la guía ARRIVE (Animal Research: Reporting of In Vivo Experiments).

## RESULTADOS

### Peso relativo del hígado

En la tabla 1, se muestra el peso promedio del hígado de pollitos donde se observan diferencias significativas (p<0,05) entre los tratamientos, con un mayor promedio para aquellos animales que recibieron manteca vegetal parcialmente hidrogenada (T2) en comparación a los demás tratamientos. 

**Tabla 1:** Peso relativo del hígado, caracterización macroscópica del hígado y morfometría intestinal de pollitos alimentados con dietas que contenían diferentes fuentes de ácidos grasos.

Variables	T1	T2	T3	T4	Valor de p
Hígado					
Peso relativo	5,19^bc^ ± 0,30	5,66^a^ ± 0,53	4,85^c^ ± 0,39	4,93^c^ ± 0,44	< 0,001
Caracterización macroscópica					
Morfometría intestinal					
Longitud (µm)	399,46^c^ ± 91,8	487,49^b^±105,02	663,71^a^ ±84,38	630,87^a^ ± 71,92	< 0,001
Ancho (µm)	83,58^ca^ ± 14,01	77,91^cb^ ± 18,01	86,77^a^ ± 16,64	74,21^b^ ± 18,92	< 0,001
Profundidad de cripta (µm)	165,83^a^ ± 177,90	131,45^a^ ±40,56	109,94^b^ ± 28,99	106,59^b^ ± 25,00	< 0,001
Área (mm^2^)	28,227^d^ ± 9,629	33,896^c^ ± 11,707	52,621^a^ ± 14,134	43,046^b^ ± 12,593	0,001
Longitud /profundidad de cripta	2,41	3,71	6,04	5,92	-

T1: 97,0% de dieta basal (DB) + 3,0% de material inerte, T2: 97,0% de DB + 3,0% de manteca vegetal parcialmente hidrogenada, T3: 97,0% de DB + 3,0% de aceite de quinua, T4: 97,0% de DB + 3,0% de aceite de pescado.

a ^-c^: Letras diferentes dentro de una misma fila evidencia diferencias significativas entre tratamientos.

Los datos fueron analizados mediante análisis de varianza (ANOVA) y Tukey para la comparación de medias entre tratamientos.

### Macroscopía y microscopía del hígado

La macroscopía del hígado revela el grado de afectación del órgano en mención, donde se puede apreciar que el hígado de los animales que recibieron el T2 (manteca vegetal parcialmente hidrogenada) muestra una coloración pálida, no brillante en casi la totalidad del órgano. En contraste, los animales que recibieron aceite de quinua o aceite de pescado presentaron características macroscópicas normales del hígado, siendo incluso similares al control (figura 1), que fueron de color rojo vino, brillo característico, ausencia de manchas. En el análisis microscópico, se pueden visualizar que los animales que recibieron la dieta T2 tuvieron una alta acumulación de gotas de grasa por efecto del consumo de la manteca vegetal parcialmente hidrogenada (figura 2), mientras que, en el grupo de animales que consumieron aceite de quinua, aceite de pescado o la dieta control, la acumulación de gotas de grasa es menor.

**Figura 1:** Análisis macroscópico del hígado de pollitos Cobb 500 alimentados con dietas conteniendo diferentes fuentes de ácidos grasos. T1: 97,0% de dieta basal (DB) + 3,0% de material inerte, T2: 97,0% de DB + 3,0% de manteca vegetal parcialmente hidrogenada, T3: 97,0% de DB + 3,0% de aceite de quinua, T4: 97,0% de DB + 3,0% de aceite de pescado.

**Figura 2:** Análisis microscópico del hígado de pollitos Cobb 500 alimentados con dietas conteniendo diferentes fuentes de ácidos grasos. T1: 97,0% de dieta basal (DB) + 3,0% de material inerte, T2: 97,0% de DB + 3,0% de manteca vegetal parcialmente hidrogenada, T3: 97,0% de DB + 3,0% de aceite de quinua, T4: 97,0% de DB + 3,0% de aceite de pescado.

### Morfometría intestinal

El tratamiento suplementado con manteca vegetal parcialmente hidrogenada (T2) obtuvo menor longitud, ancho y área del vello respecto a ambos aceites (T3 y T4) y mayor profundidad de cripta en comparación a T3 y T4 (p<0,05) y un valor similar a la dieta basal. La suplementación con aceite de quinua (T3) tuvo mayor valor en longitud, ancho y área del vello intestinal, pero menor valor en profundidad de cripta; seguido muy de cerca por el tratamiento que recibió aceite de pescado (T4; p<0,05), que reportó valores similares en longitud y profundidad de cripta pero menores para el ancho y área de vello.

La relación entre la longitud del vello intestinal y profundidad de la cripta en los tratamientos suplementados con aceite de quinua (T3: 6,04) o aceite de pescado (T4: 5,92) tuvieron valores superiores para este indicador, en comparación a aquellos que consumieron manteca vegetal parcialmente hidrogenada (T2: 3,71).

## DISCUSIÓN

Resulta interesante conocer el efecto de las fuentes de grasa sobre las características del hígado y la morfometría del intestino delgado en un modelo animal durante la edad temprana. El hígado es el sitio central donde se produce la lipogénesis *de novo*, que es una forma de comunicación cruzada entre el órgano y los lípidos con la finalidad de mantener la homeostasis metabólica ^17^. En la investigación, se determinó que los animales que consumieron manteca vegetal parcialmente hidrogenada (T2) tuvieron un mayor peso relativo del hígado y un grado de afectación I (o leve), que sugiere el inicio de un posible hígado graso; esto se debería probablemente al aumento de la lipogénesis ^18^^)^ y la presencia de AGS en la dieta que se asocian con una mayor acumulación de triglicéridos intrahepáticos ^19^. El mecanismo sugerido, se basa en que el consumo de AGS incrementa la inflamación en el tejido adiposo, y consecuentemente, promueve la lipolisis de este tejido, conduciendo a un mayor flujo de ácidos grasos (dietéticos y de tejido adiposo) al hígado e incrementando las ceramidas, las cuales al parecer causan resistencia a la insulina, lo que conduciría a la acumulación de triglicéridos intrahepáticos ^19^. Incluso, estudios previos refieren que la influencia del consumo elevado de AGS sobre el desarrollo de inflamación en el hígado, puede agravarse si el consumo de estos se torna continuo, lo que promovería al desarrollo de hígado graso ^7^^,^^20^. En contraste, existe evidencia que el consumo de AGI (ω3 y ω6) disminuye y/o previene la acumulación de triglicéridos intrahepáticos en comparación a los AGS ^19^.

El contraste con el análisis microscópico, indica que aquellos animales que consumieron aceite de quinua (T3), aceite de pescado (T4) o la dieta control (T1), tuvieron menor acumulación de grasa debido al efecto de los AGPI sobre la β-oxidación; asimismo, estos también influyen en el aumento de adipocinas antinflamatorias como la Interleucina-10, que está ampliamente relacionado con el síndrome metabólico por su capacidad de inhibir la síntesis de citocinas inflamatorias ^21^. Incluso, se ha determinado que el consumo de aceites a base de palma al 10% incrementa la actividad en los vasos sanguíneos del hígado promoviendo la inflamación de este órgano, pero es posible notar que en el presente estudio, la ingesta de la dieta T2, que contenía manteca vegetal parcialmente hidrogenada al 3%, generó afecciones a este órgano ^22^.

En la morfometría intestinal, la relación longitud/profundidad de cripta evidencia que el consumo de aceite de quinua (T3) seguido del aceite de pescado (T4), promueven valores superiores que se relacionan directamente con una mejora en la digestión y absorción de nutrientes debido a su contenido de AGPI ^23^^,^^24^, en comparación al consumo de manteca vegetal parcialmente hidrogenada (T2) y la dieta basal (T1). Algunas investigaciones sugieren que, la composición de ácidos grasos modula primero, la tasa de digestión intestinal y luego los niveles de los lípidos séricos ^25^, e incluso el consumo de aceites vegetales a un nivel ≥6% afecta la absorción intestinal y promueve la formación de una capa submucosa delgada, lo que reduciría la expansión del intestino cuando se ingiere un gran volumen de comida ^26^. Por otro lado, se ha evidenciado que el consumo de aceites ricos en AGPI puede atenuar el transporte de endotoxinas, en contraste, la ingesta de AGS incrementa el transporte de los mismos ^27^.

Entre las limitaciones debemos mencionar que, no se realizaron análisis más específicos para determinar si hubo o no desarrollo de hígado graso en el grupo que recibió manteca vegetal parcialmente hidrogenada. Asimismo, si bien el modelo animal utilizado acumula grasa en un depósito similar al humano, no es posible inferir dicha respuesta, pero si resulta necesario investigar al respecto debido a los hallazgos y su relación con la salud.

Por todo lo mencionado, el aceite de quinua roja constituye una fuente grasa beneficiosa por su capacidad de favorecer la formación de adipocitos relacionados con un tejido adiposo saludable en el organismo, siendo esta investigación una base para futuros estudios sobre su impacto en la función metabólica de otros órganos y la salud, así como, su potencial como ingrediente en la elaboración de productos de consumo humano con la finalidad de beneficiar a la salud de la población.

En conclusión, el consumo de las fuentes de grasa tales como, aceite de quinua y aceite de pescado redujo el peso relativo y mantuvo adecuadas características macroscópicas-microscópicas del hígado, además, favoreció una mejor morfometría del intestino en el modelo animal de temprana edad. Por el contrario, la manteca vegetal parcialmente hidrogenada generó un incremento en el peso del órgano, alteración en su estructura macroscópica-microscópica y menor capacidad de absorción a nivel intestinal. Lo que sugiere, que el aceite de quinua posee gran potencial para ser incluido dentro de la dieta para consumo humano.
